# MicroR159 regulation of most conserved targets in *Arabidopsis *has negligible phenotypic effects

**DOI:** 10.1186/1758-907X-1-18

**Published:** 2010-10-28

**Authors:** Robert S Allen, Junyan Li, Maria M Alonso-Peral, Rosemary G White, Frank Gubler, Anthony A Millar

**Affiliations:** 1 Research School of Biology, Australian National University, Canberra, Australian Capital Territory, Australia; 2 CSIRO Plant Industry, Canberra, Australian Capital Territory, Australia; 3 School of Molecular Bioscience, University of Sydney, New South Wales, Australia

## Abstract

**Background:**

A current challenge of microRNA (miRNA) research is the identification of biologically relevant miRNA:target gene relationships. In plants, high miRNA:target gene complementarity has enabled accurate target predictions, and slicing of target mRNAs has facilitated target validation through rapid amplification of 5' cDNA ends (5'-RACE) analysis. Together, these approaches have identified more than 20 targets potentially regulated by the deeply conserved miR159 family in *Arabidopsis*, including eight *MYB *genes with highly conserved miR159 target sites. However, genetic analysis has revealed the functional specificity of the major family members, miR159a and miR159b is limited to only two targets, *MYB33 *and *MYB65*. Here, we examine the functional role of miR159 regulation for the other potential *MYB *target genes.

**Results:**

For these target genes, functional analysis failed to identify miR159 regulation that resulted in any major phenotypic impact, either at the morphological or molecular level. This appears to be mainly due to the quiescent nature of the remaining family member, *MIR159c*. Although its expression overlaps in a temporal and spatial cell-specific manner with a subset of these targets in anthers, the abundance of miR159c is extremely low and concomitantly a *mir159c *mutant displays no anther defects. Examination of potential miR159c targets with conserved miR159 binding sites found neither their spatial or temporal expression domains appeared miR159 regulated, despite the detection of miR159-guided cleavage products by 5'-RACE. Moreover, expression of a miR159-resistant target (*mMYB101*) resulted predominantly in plants that are indistinguishable from wild type. Plants that displayed altered morphological phenotypes were found to be ectopically expressing the *mMYB101 *transgene, and hence were misrepresentative of the *in vivo *functional role of miR159.

**Conclusions:**

This study presents a novel explanation for a paradox common to plant and animal miRNA systems, where among many potential miRNA-target relationships usually only a few appear physiologically relevant. The identification of a quiescent miR159c:target gene regulatory module in anthers provides a likely rationale for the presence of conserved miR159 binding sites in many targets for which miR159 regulation has no obvious functional role. Remnants from the demise of such modules may lead to an overestimation of miRNA regulatory complexity when investigated using bioinformatic, 5'-RACE or transgenic approaches.

## Background

MicroRNAs (miRNAs) regulate gene expression by guiding the RNA induced silencing complex (RISC) to gene targets via base pairing complementarity [1]. For most plant miRNAs, their target mRNAs contain motifs that have perfect/near perfect complementarity resulting in a regulatory mechanism that includes RISC-directed slicing [2]. Due to these high sequence complementarity requirements, it has been relatively easy to bioinformatically predict potential targets for a particular miRNA in plants [3]. For ancient miRNAs, conservation of target motifs over long evolutionary distances have further aided in the identification of targets, and highlighted the importance of the miRNA:target interaction [2]. Moreover, a hallmark of high complementarity miRNA-mediated regulation has been the isolation of miRNA-guided target mRNA cleavage products by rapid amplification of 5' complementary DNA ends (5'-RACE) methods [4]. The detection of these products designates such mRNAs as experimentally validated miRNA targets [5-7]. This has now been extrapolated onto a genome wide scale, where the sequencing of plant degradomes has identified many miRNA targets [8-10]. With the recent proliferation of genomic data from many plant species, bioinformatics and degradome data will be at the forefront of predicting and identifying a diverse array of new miRNAs and their targets with a high degree of confidence [11,12].

However, perhaps a more important but overlooked question relates to the identification of miRNA:target relationships that are biologically relevant [13]. Although the commonly taken approaches described above have validated many miRNA:target interactions, the *in vivo *relevance of such interactions is often difficult to assess. Notably, in the few plant and animal miRNA studies where specific miRNAs have been removed or mutated, most phenotypic abnormalities can be attributed to deregulation of a discrete subset of targets [14-18]. This apparent paradox is hard to reconcile with an abundance of bioinformatic and molecular data that argues many validated targets may exist for a particular miRNA.

This also applies for the miR159 family in *Arabidopsis *[14]. This family has three different members, miR159a, miR159b and miR159c [19,20], and represents one of the most ancient miRNAs in the plant kingdom [21], and it is also the most abundant miRNA family in *Arabidopsis *[22-24]. Extensive bioinformatic and molecular analysis predicts this family has potentially 20 or more target genes (Additiona file1). This includes eight genes encoding R2R3 MYB domain proteins that have miR159 binding sites that are strongly conserved in both monocot and dicot species. The miR159 family is also related to the miR319 family [19,20], yet sequence differences prevent miR159 regulating targets of miR319 [25], while the spatial expression domains and low abundance of miR319 family members prevents major regulation of miR159 targets [25].

To determine the functional role of miR159 regulation, loss-of-function mutations in the two predominantly expressed miR159 members, miR159a and miR159b, were obtained. Functionally redundancy was demonstrated, as a *mir159ab *double mutant displayed strong pleiotropic developmental defects not apparent in either single mutant [14]. However only two targets, *MYB33 *and *MYB65 *were deregulated in *mir159ab*. The biological importance of this relationship was implicitly demonstrated by suppression of all pleiotropic *mir159ab *phenotypes in a quadruple *mir159ab/myb33/myb65 *mutant, implying that miR159a/miR159b are functionally specific for only 2 of the 20 predicted/validated target genes [14]. This specificity was reconciled by the observation that many targets appear to have mutually exclusive transcriptional domains when compared to that of miR159a/b; they were transcribed predominantly in anthers where the near ubiquitous miR159a/b appeared absent. Therefore *MYB33 *and *MYB65 *would be considered switch targets, whereas the other targets would be considered neutral targets [26].

This raises the question of what is the selective pressure driving miR159 target site conservation in these other potential target genes. Confounding this, multiple lines of evidence exist suggesting the other targets are regulated by miR159, including five genes that have been validated by 5'-RACE (Additional file 1; [25,27,28]). For instance, *MYB101*, the closest related gene to *MYB33*/*MYB65*, contains a highly conserved miR159 binding site, is downregulated in flowers of *35S:MIR159a Arabidopsis *plants [29] and miR159-guided cleavage products corresponding to *MYB101 *have been isolated validating it as a genuine miR159 target [25,27,28].

There are numerous possibilities for the requirement of miR159-binding sites in these other potential targets. Firstly, they may be regulated by the third member of the family, miR159c. Although lowly abundant due to probable poor processing [25], deep sequencing indicates miR159c is still expressed [30], and at similar levels to other miRNAs, such as miR164c, which is required for proper floral development [31]. Secondly, like *myb33.myb65 *[32], the *mir159ab*/*myb33.myb65 *mutant was male sterile that may mask any potentially important miR159 regulation in the anther. This is important as of the 20 or so potential miR159 targets, 12 of them are expressed in anthers/pollen or associated with their development (Additional file 1). Another possibility is that *mir159ab *may only be a hypomorphic mutant, able to produce miR159 at levels sufficient to silence most target genes, but not at high enough levels to fully suppress *MYB33 *and *MYB65*. Alternatively, environmental conditions may exist where miR159 is induced, repressing these other target genes. This could include the possibility that miR159 acts as a safeguard against these anther transcribed genes, attenuating 'leaky' transcription that may occur in other tissues, a theme that is common in animals [33].

From exploring these possibilities, our analyses suggest that miR159c has subfunctionalised and corresponds to a distinct anther miR159c:target gene regulatory module. However in *Arabidopsis *the activity of *MIR159c *is so weak it has negligible impact on its potential target genes. Thus the remnants of this miR159c regulatory module may provide an explanation for the presence of conserved miRNA target sites in genes that appear principally independent of miR159 regulation. Our study highlights the need for loss-of-function analysis in identifying key miRNA:target relationships and that in fact other methodologies may be overestimating the scope and extent of miRNA regulation.

## Results

### A *mir159abc *triple mutant appears indistinguishable from *mir159ab*

**Figure 1:**
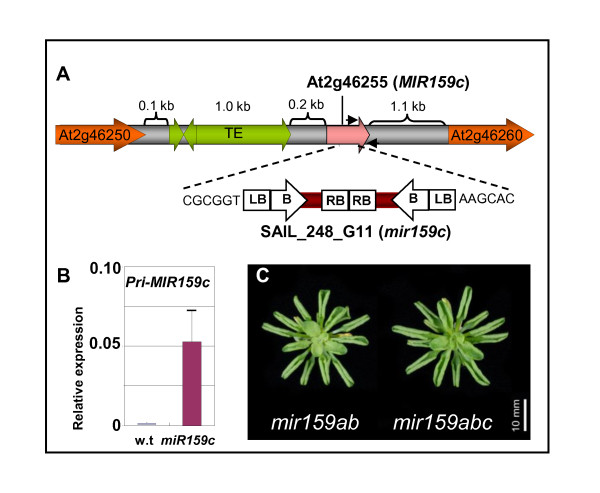
**A potential null allele of *MIR159c *does not result in any developmental or molecular alterations**. **(A) **The genomic context of the *MIR159c *(At2g46255) gene showing the position of transposable elements (TE, green) that are located 214 bp upstream of the stem-loop region (pink). The insertion site of the transfer DNA (T-DNA) in the SAIL_248_G11 (*mir159c*) line is indicated by the dashed line. Large arrows indicate the location and direction of the transcriptional units, B = basta resistance gene; LB = left border; RB = right border. **(B) **Real-time quantitative (qRT)-PCR on inflorescences of wild-type and *mir159c *plants. The approximate positions of the primers used for qRT-PCR are indicated with small black arrows in (A). Expression values were normalised to cyclophilin, with measurements being the average of three replicates and error bars representing the standard error of the mean. **(C) **Rosette phenotypes of short-day grown *mir159ab *and *mir159abc*.

The only annotation corresponding to the *MIR159c *gene (At2g46255) is a 225 bp putative pre-miR159c stem-loop structure located in an intergenic region on chromosome 2. There is a cluster of three transposable elements located only 214 bp upstream of this pre-miR159c sequence (Figure 1a), which is absent from this locus in the *Arabidopsis lyrata *genome [34,35], indicating that these insertions are relatively recent events. Based on the structures and sizes of the *MIR159a *and *MIR159b *genes [14], these elements would be predicted to be located in the 5' promoter/*pri-*transcript region of *MIR159c*, possibly affecting its activity.

To determine the functional importance of *MIR159c*, the transfer DNA (T-DNA) insertional mutant SAIL_248_G11 was obtained from the SIGnAL database [36], hereafter designated *mir159c*. Isolation and sequencing of both T-DNA junctions found the T-DNA had integrated in an inverse tandem manner within the stem-loop region (Figure 1a). Although endogenous levels of *pri-MIR159c *are extremely low, elevated *pri-mir159c *transcript was detected in *mir159c *(Figure 1b), suggesting that the T-DNA is affecting the transcription and/or processing of this gene. Given the location of the T-DNA between the miR159c and miR159c* sequences, any transcript from this allele would be unlikely to form the secondary structure required to enable processing into mature miR159c and hence *mir159c *is most likely a null allele. Despite this, the morphological phenotype of *mir159c *was indistinguishable from wild type and a *mir159abc *triple mutant was indistinguishable from *mir159ab *(Figure 1c), the latter suggesting that no additional redundancy between miR159a/b and miR159c exists.

### *mir159abc *represents a very strong loss-of-function miR159 mutant

Next, we determined the precise extent to which miR159 has been downregulated in *mir159abc*. Previously northern blotting of *mir159ab *using a miR159a probe failed to detect expression of miR159 in *mir159ab *[14], however the *mir159a *allele may still retain some activity as the T-DNA had inserted outside of the stem-loop region, whereas *mir159b *and *mir159c *were assumed to be null alleles because of the stem-loop location of the T-DNA. Because the sequences of the three miR159 members are highly similar (Additional file 1), conventional northern blotting would not be expected to easily differentiate between the three family members. Therefore we used ABI TaqMan microRNA quantitative real-time stem-loop PCR assays (qSL-PCR; http://www.appliedbiosystems.com/) to assess the miR159 levels in all three *mir159 *mutants.

**Figure 2:**
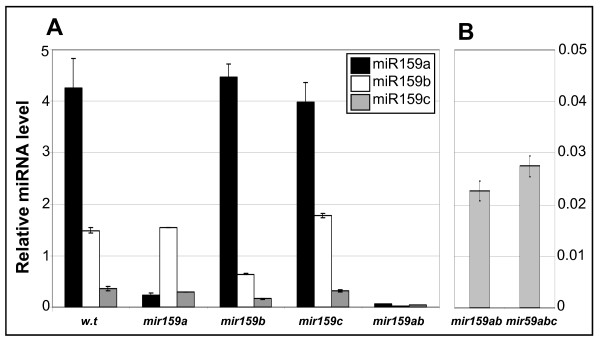
**TaqMan microRNA assay measurement of mature miR159 isoforms in wild type and the various *mir159 *mutants**. Analysis was performed on RNA extracted from inflorescences, and miRNA abundance was normalised to *sno101 *with measurements being the average of three replicates with error bars representing the standard error of the mean. **(A) **Measurement of miR159a, miR159b and miR159c in wild type, the three single *mir159 *mutants and *mir159ab*. **(B) **Measurement of miR159c in *mir159ab *and *mir159abc*.

Firstly, to validate the use of qSL-PCR for miR159 quantification, RNA was prepared from an analogous wild-type sample (same ecotype (Columbia), day length (16 h day) and tissue (inflorescences)) on which previous deep sequencing found the relative abundance of miR159a, miR159b and miR159c to be 87.2%, 12.6% and 0.2%, respectively [30]. Analysis using the qSL-PCR assays found the relative abundance of miR159a, miR159b and miR159c to be 69.7%, 24.4% and 5.9%, respectively (Figure 2a). Although the trend in the relative abundance of the different members correlated with the deep sequencing data, the absolute percentages varied considerably between the data sets. Although this may in part reflect biological variation, crossreaction with different miR159 members could also be a contributing factor.

To assess this, assays were performed on the single *mir159 *mutants (Figure 2a). This revealed the assays generally had high, but not absolute specificity. For example, comparison of miR159a levels in *mir159a *(0.24) with *mir159ab *(0.06) indicates the miR159a TaqMan assay crossreacts with miR159b in *mir159a*. Similarly, the levels of miR159c found in wild type (0.36) and *mir159c *(0.32) are likely representative of the miR159c assay crossreacting with miR159a and miR159b, as demonstrated by the level of miR159c in *mir159ab *(0.04). Therefore to accurately measure the reduction of miR159 members in *mir159a*, *mir159b *and *mir159c*, a comparison between wild type, *mir159ab, *and *mir159abc *was required. It was found both mutant alleles of *mir159a *and *mir159b *were expressing their respective miR159 products to less than 1% of wild-type levels (Figure 2a). For *mir159b*, this confirms that the mutant is a likely null allele [14]. For *mir159a*, the low level of miR159a from this locus was somewhat surprising, given that the T-DNA insertion was outside the stem-loop region and only reduced *pri-mir159a *levels sixfold [14], suggesting this T-DNA insertion reduces both transcription and processing of *pri-MIR159a*, leading to what would be considered a strong loss-of-function *mir159a *allele. Therefore, *mir159ab *does not represent a miR159 hypomorphic mutant, but instead is a strong loss-of-function mutant. For *mir159c*, assays were performed on *mir159ab *and *mir159abc *and no differences in the level of miR159c between the two genotypes could be found (Figure 2b), demonstrating the level of miR159c measured in inflorescences is so low that it is beyond the detection limits of qSL-PCR analysis.

### A *35S*:*MIR159c *transgene is a potent silencer of *MYB33 *and *MYB65*

**Figure 3:**
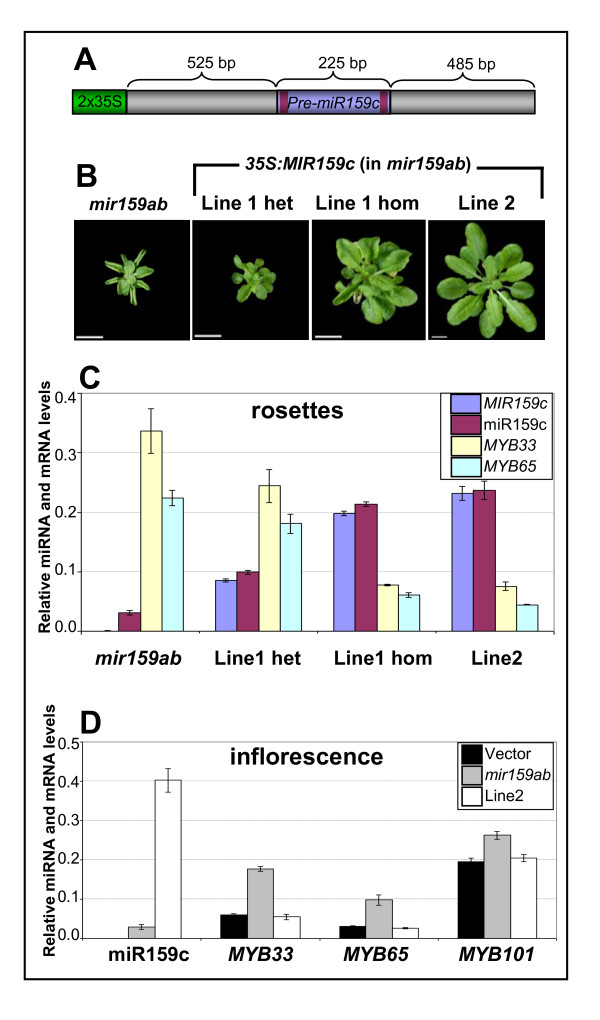
**A *35S:MIR159c *transgene can complement *mir159ab***. **(A) **The *35S:MIR159c *transgene with relevant regions shown. The dark purple bars represent miR159c* and miR159c sequences of *MIR159*c. 2X35S = tandem 35S promoter of the plasmid vector pMDC32. Figure is not to scale. **(B) **Rosette phenotypes of transgenic *mir159ab *lines transformed with the *35S:MIR159c *construct. For line 1, both heterozygous and homozygous segregants are shown. **(C) **Analysis of *MIR159c *transcript and mature miR159c, and *MYB33 *and *MYB65 *expression in *35S:MIR159c *(*mir159ab*) transgenic rosettes. **(D) **Analysis of mature miR159c and *MYB *levels in; wild type transformed with empty (vector), *mir159ab *and *35S:MIR159c *(*mir159ab*) line 2 inflorescences. Measurement of miR159c is not shown in the empty vector line due to crossreaction of the assay with miR159a and miR159b (Figure 2), which overstates the absolute abundance of miR159c. Measurements represent the average of three replicates with error bars showing the standard error of the mean. mRNA levels were normalised to cyclophilin and miR159c abundance was normalised to *sno101*.

The very low miR159c expression level and wild-type phenotype of *mir159c *suggested that *MIR159c *may be a pseudogene. To determine if transcripts from this locus have any potential activity, a *35S:MIR159c *transgene (Figure 3a) was generated. Unlike a *35S:MIR159c *construct reported previously that used only the *pre-MIR159c *sequence [25], we also included additional flanking sequences for our *35S:MIR159c *transgene that could be important for processing efficiency of the mature miRNA. The *35S:MIR159c *construct was transformed into either wild-type or *mir159ab *plants. All of the 20 *35S:MIR159c *(wild-type) plants generated were morphologically indistinguishable from wild type, and none displayed male sterility, the characteristic phenotype of *35S:MIR159a *transgenic *Arabidopsis *[29,37]. This is in agreement with the finding that *MIR159c *has less activity than *MIR159a *even when expressed under similar promoters [25]. However the *35S:MIR159c *construct was sufficiently active to complement the *mir159ab *mutant. Of 10 *35S:MIR159c *(*mir159ab*) transformants analysed, 8 were partially or fully complemented (Figure 3b), implying that miR159c can repress *MYB33 *and *MYB65 *expression as predicted [25]. Consistent with this, repression of *MYB33 *and *MYB65 *transcript levels correlated tightly with increased levels of *MIR159c *precursor and mature miR159c (Figure 3c). Moreover, as quantified by the qSL-PCR assays, miR159c only needed to be expressed at approximately 4% of total wild-type miR159 levels for complementation to occur (Figure 3b,c). This is supported by genetic analysis that indicates only small quantities of miR159 are required to fully repress *MYB33 *and *MYB65*; only the presence of a single allele of either *MIR159a *or *MIR159b *results in a morphologically wild-type plant [14]. Therefore, like miR159a and miR159b, miR159c is a potent silencer of *MYB33 *and *MYB65 *when expressed at high enough levels. Based on these observations, *MIR159c *could not be regarded as a pseudogene. However the *35S*:*MIR159c *transgene was unable to induce male sterility (Additional file 2, Figure S1), even in the *mir159ab *transformed line that showed the highest miR159c levels (line 2) (Figure 3d). This may be indicative of the high steady state mRNA levels of these *MYB *genes in anthers in comparison to rosettes, or it could hint that in some tissues, miR159 activity is enhanced or attenuated leading to different regulatory outcomes.

### Expression of *MIR159c *is limited to a narrow range of cell types

**Figure 4:**
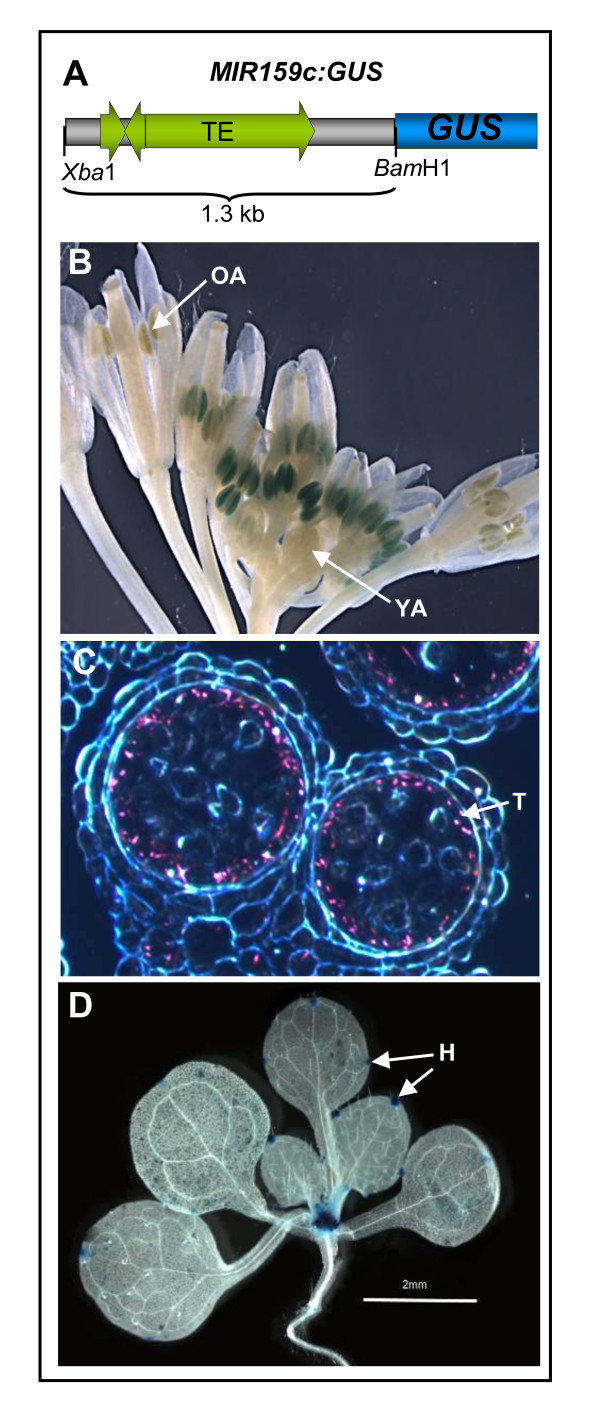
**Expression of a *MIR159c*:*GUS *transgene is restricted to specific cell types**. **(A) **Diagram showing the sequences used for construction of the *MIR159c*:*GUS *transgene in the vector pBI101.1. **(B) **β-Glucuronidase (GUS)-stained *MIR159c*:*GUS *inflorescences. OA = old anthers; YA = young anthers. **(C) **Dark field microscopy of a transverse section of a *MIR159c*:*GUS *anther. GUS staining is shown by pink crystals. T = tapetum. **(D) **GUS staining (48 h) in a 14-day-old *MIR159c*:*GUS *plant. H = hydanthodes.

A possible explanation for the low abundance of miR159c and the absence of a distinguishable *mir159c *phenotype is that miR159c may be restricted to a specific set of cells. This would be in contrast to miR159a and miR159b that appear broadly expressed throughout the plant, but notably absent in anthers [14]. Therefore to determine in which cells *MIR159c *is transcribed, a *MIR159c:GUS *construct was generated that used sequences 1.3 kb immediately upstream of the *MIR159c *stem-loop region, including all intergenic sequences (with transposable elements) to the next upstream protein-coding gene (Figure 4a).

Strikingly, the expression pattern of *MIR159c*:*GUS *in inflorescences appeared reciprocal to the *MIR159a*:*GUS *and *MIR159b*:*GUS *transgenes, being expressed only in anthers (Figure 4b). This β-glucuronidase (GUS) expression was temporally controlled, being absent in young anthers, but visible in floral stages 6-12 and then weakening again in mature anthers. Transverse sections of anthers found staining was restricted to the tapetal cell layer in postmeiotic anthers, with staining strongest during the microspore stage of development (Figure 4c). Degeneration of the tapetum coincided with the loss of staining, hence explaining the transient nature of expression in anthers. In vegetative tissues, the *MIR159c*:*GUS *transgene was specifically expressed in hydathodes and in the shoot apical region with no other cell type showing any staining (Figure 4d). Restriction of *MIR159c *expression to these specific cell types provides part of the explanation for the low abundance of miR159c when compared to the broadly transcribed *MIR159a *and *MIR159b *genes [14].

### *MYB33 *5'-RACE product abundance appears higher than other miR159 targets

As miR159a and miR159b are functionally specific for *MYB33*/*MYB65*, and *mir159c *does not display a mutant phenotype, the question arose as to whether the other *MYB *target genes with conserved miR159 binding sites were in fact miR159 regulated. As many of these *MYB *genes are predominantly transcribed in anthers and/or pollen (Additional file 1), 5'-RACE cleavage assays of genes using RNA isolated from inflorescence tissues was carried out. Furthermore to gain insight into what extent miR159c-guided cleavage may be involved in regulating these *MYB *genes, this analysis was also performed on *mir159ab*. To ensure that the integrity of the 5'-RACE cDNA was equivalent between wild type and *mir159ab*, PCR using *MYB101 *and *MYB81 *primers annealing downstream of the 5'-RACE adapter showed similar levels of amplification (Additional file 3, Figure S2).

**Figure 5:**
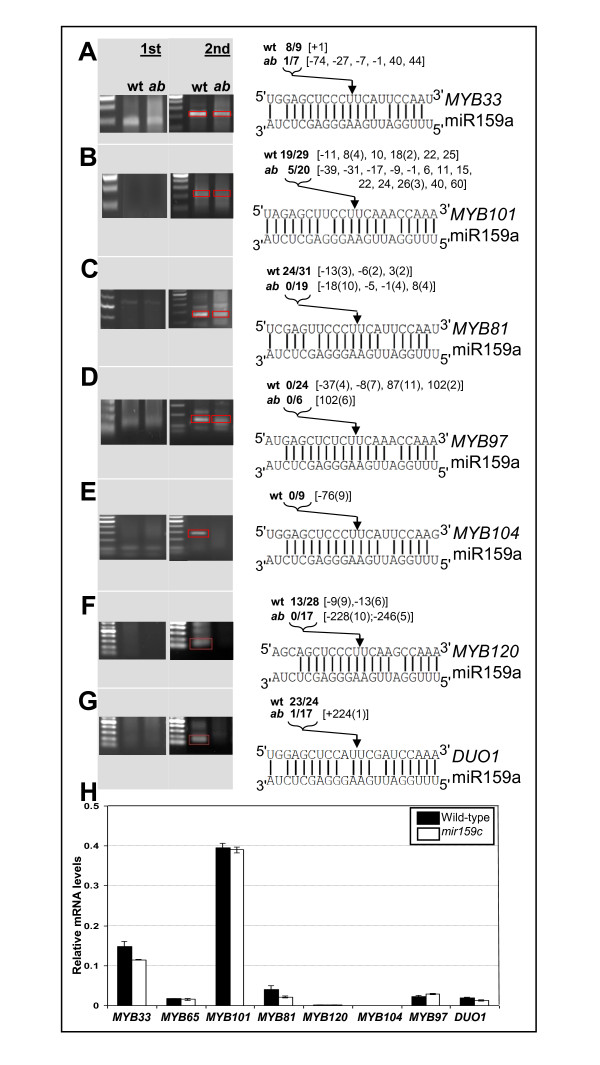
**miR159 cleavage assays of potential target genes**. Inflorescence purified mRNA from wild type and *mir159ab *was ligated with rapid amplification of 5' complementary DNA ends (5'-RACE) adapters and subject to 5'-RACE PCR. The products from first (1st) and nested (2nd) rounds of PCR were analysed by agarose gel electrophoresis. DNA from the nested PCR reactions was either directly cloned or the expected size PCR products gel purified before cloning (indicated by red box). Clones were then sequenced and mapped. The sequence similarity of each target is shown compared to miR159a. Numbers in bold indicate the proportion of clones out of the total number analysed that mapped to the canonical miR159 cleavage position (indicated by arrow) either in wild type (wt) or *mir159ab *(ab). Numbers inside square brackets indicate the position of any further clones relative to the miR159 cleavage site, with (-) numbers indicating fragments that map upstream of the miR159 cleavage site and (+) numbers indicating fragments that are further downstream of the miR159 cleavage site. Numbers in round brackets indicate the number of multiple clones found at that position. Analysis was carried out for: **(A) ***MYB33*, **(B) ***MYB101*, **(C) ***MYB81*, **(D) ***MYB97*, **(E) ***MYB104*, **(F) ***MYB120 *and **(G) ***DUO1 *(*MYB125*). **(H) **Real-time quantitative (qRT)-PCR analysis of mRNA abundance of putative miR159c target genes in wild type (black bars) and *mir159c *(white bars). Analysis was performed on RNA extracted from inflorescences with measurements being the average of three replicates with error bars representing the standard error of the mean.

In wild-type inflorescences, miR159-guided cleavage products were found for *MYB33*, *MYB101 *and *DUO1 *(Figure 5a, b, g), as has been previously reported [25,27] and also *MYB81 *and *MYB120 *(Figure 5c, f). No cleavage products could be found for *MYB97 *or *MYB104 *even after using several different 5'-RACE primer combinations (Figure 5d, e). Of all these genes, 5'-RACE for only *MYB33 *produced a visible PCR product of the expected size after the first round of PCR (Figure 5a). Although 5'-RACE assays are inherently non-quantitative, the presence or absence of 5'-RACE PCR products correlates closely with the notion that miR159 regulation of *MYB33 *(and *MYB65*) is much more extensive than it is for the other *MYB *target genes, as determined by genetic analysis and transcript profiling [14]. Moreover, degradome data readily found *MYB33 *and *MYB65 *cleavage products, while cleavage products for the other *MYB *targets were not identified [9]. Also 5'-RACE products not corresponding to the miR159 cleavage site were cloned multiple times at identical positions for *MYB81*, *MYB97*, *MYB104*, *MYB120 *and *DUO1 *(Figure 5), suggesting that only a few transcripts of each gene were being assayed, highlighting the sensitivity of this method, which may only be detecting basal level regulation.

miR159-guided cleavage products for *MYB33*, *MYB101*, *MYB81*, *MYB120 *and *DUO1 *were cloned with far lower frequency from *mir159ab *5'-RACE cDNA. This suggests that cleavage of these *MYB *genes is carried out primarily by miR159a/miR159b. Consistent with this, mRNA abundance of these *MYB *genes was unchanged in the inflorescences of *mir159c *when compared to wild type (Figure 5h), demonstrating that the levels of miR159c present in wild-type anthers and any slicing it performs is insufficient to have an impact on mRNA abundance of these target genes.

Finally, the 5'-RACE assays are also able to detect any miR319 regulation, as miR319 guides cleavage one nucleotide upstream of the miR159 cleavage position [25]. miR319-guided cleavage only composed a very minor amount of cleavage events and these were only detected in the absence of miR159a and miR159b, suggesting that none of these *MYB *genes are under strong miR319 regulation. This is also consistent with previous findings [25].

### Pollen development and germination appears independent of miR159 regulation

**Figure 6:**
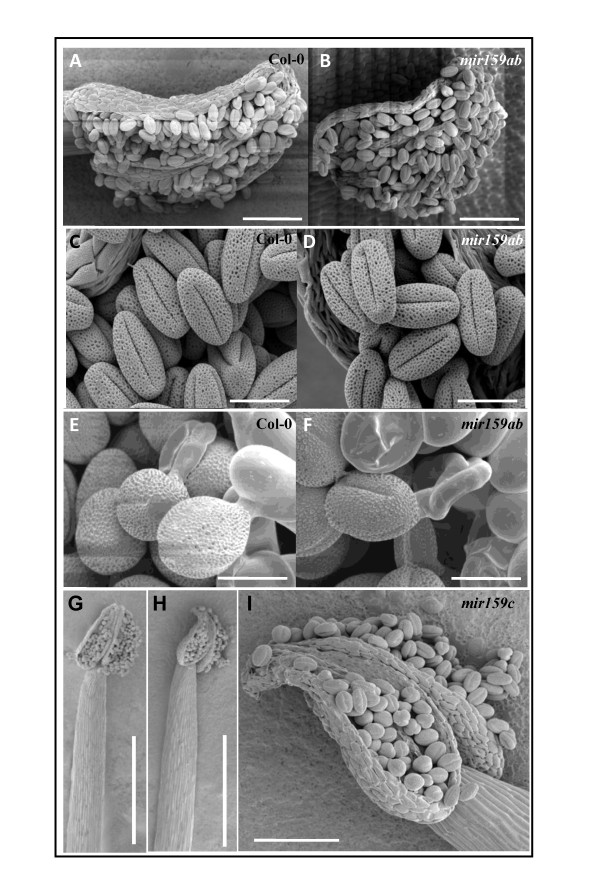
**Wild-type and *mir159ab *pollen grains are indistinguishable**. Scanning electron microscope (SEM) images of wild-type **(A,C,E,G) **and *mir159ab ***(B,D,F) **anthers, pollen grains and germinating pollen grains, respectively. **(H,I) **SEM images of miR159c anthers and pollen. Scale bars represent 100 (A,B,I), 20 (C-F) and 500 (G,H) μm.

To address whether any of the miR159:*MYB *target genes relationships has any developmental consequence in inflorescences, we examined anthers and pollen of the *mir159 *mutants, tissues in which these *MYB *target genes are strongly (and/or predominantly) transcribed and in which miR159 is present [38]. However there were no obvious morphological differences between wild-type and *mir159ab *mature or germinating pollen (Figure 6a-f). Consistent with this, analysis found the *mir159a *and *mir159b *alleles segregated in the expected Mendelian ratio (521 wild type: 32 *mir159ab*, 1:15, χ^2 ^= 0.20, *P *= 0.65) from a F1 *mir159a*/*mir159b *heterozygote [14] indicating that the *mir159a *or *mir159b *alleles have no obvious quantitative effect on pollen viability or germination in the context of competing with wild-type pollen grains. For *mir159c*, again there was no obvious alteration of anther or pollen morphology (Figure 6g-i), consistent with the finding that *MIR159c *has negligible activity in anthers.

### MYB101 or MYB120 expression in anthers is not delineated by miR159

**Figure 7:**
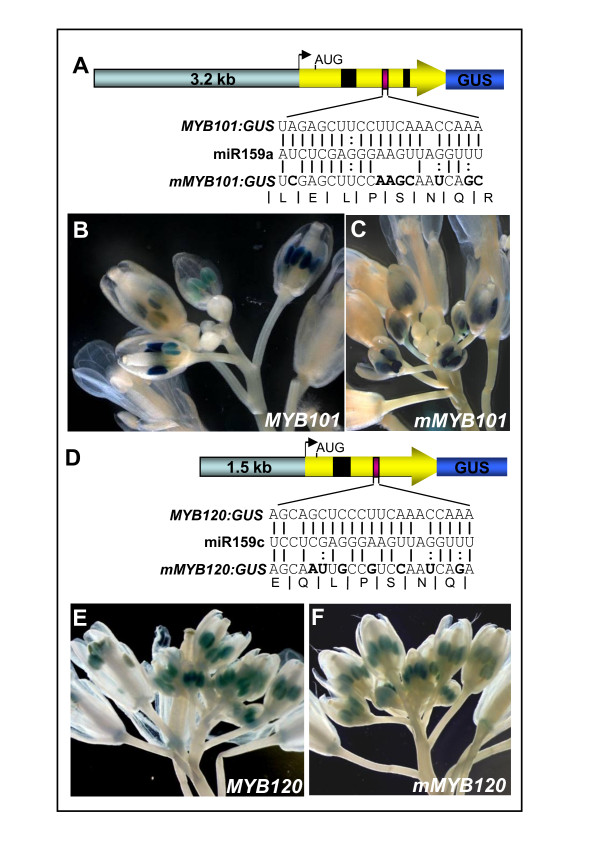
**Expression of *MYB101 *and *MYB120 *is not regulated by miR159 in inflorescences**. **(A) **The *MYB101*:*GUS *transgene consisted of 5171 bp of genomic sequence that included, 3.2 kb of 5' flanking region extending to the adjacent upstream gene (At2g32470), and 1.9 kb of the transcribed region of *MYB101 *(yellow arrow) that includes introns (black boxes) and the miR159 binding site (purple box) fused in frame to the β-glucuronidase (GUS) reporter gene to encode a full length MYB101:GUS translational fusion protein. Eight synonymous nucleotide substitutions were made in the miR159 binding site of *MYB101:GUS *to generate *mMYB101:GUS*. Figure is not to scale. **(B) **GUS staining of the inflorescence of a *MYB101*:*GUS *transgenic plant. **(C) **GUS staining of the inflorescence of a *mMYB101*:*GUS *transgenic plant. **(D) **The *MYB120*:*GUS *transgene consisted of 3125 bp of genomic sequence that included, 1.5 kb of 5' flanking region and 1.6 kb of the coding region of *MYB120 *(yellow arrow) that includes an intron (black box) and the miR159 binding site (purple box) fused in frame to the GUS reporter gene to encode a full length MYB120:GUS translational fusion protein. Seven synonymous nucleotide substitutions were made in the miR159 binding site of *MYB120:GUS *to generate the *MYB120:GUS *transgene. Figure is not to scale. **(E) **GUS staining of the inflorescence of a *MYB120*:*GUS *transgenic plant. **(F) **GUS staining of the inflorescence of a *mMYB120*:*GUS *transgenic plant.

Next we functionally analysed the extent of miR159 regulation for two target genes, *MYB101 *and *MYB120*, both of which have highly conserved miR159 binding sites. Firstly, as the preceding analysis has not addressed the possibility of translational repression, *MYB:GUS *translational fusions were generated. To maximise the likelihood of faithfully reproducing endogenous expression, the *MYB101:GUS *and *MYB120:GUS *(miRNA regulated) transgenes contained extensive 5' flanking sequences and the entire coding regions (Figure 7a, d). To generate miRNA-resistant versions, site-directed mutagenesis was used to make eight (*mMYB101:GUS*) and seven (*mMYB120:GUS*) nucleotide changes to disrupt miRNA regulation while conserving the wild-type protein coding sequences (Figure 7a, d). Other than the conserved miR159 target sites in *MYB101 *and *MYB120*, there were no other potential miR159 target sites present in these transgenes. They were transformed into *Arabidopsis *allowing any miRNA regulation of *MYB101 *and *MYB120 *expression to be visualised.

The majority of *MYB101:GUS *(6/10) and *mMYB101:GUS *(7/8) lines showed anther-specific GUS expression in the inflorescence (Figure 7b, c), a pattern indistinguishable from *proMYB101*:*GUS *(*MYB101 *promoter only) transgenic plants [14] and consistent with online microarray data showing *MYB101 *is overwhelmingly expressed in anthers and pollen [39]. At the gross organ level, there was no evidence of an expanded spatial or temporal *mMYB101*:*GUS *expression domain (Figure 7b, c). Expression of both the *MYB101*:*GUS *and *mMYB101*:*GUS *transgenes appeared restricted to anthers of intermediate age (approximately floral stages 6-12) but absent in both younger and older anthers. This spatial and temporal expression pattern appears highly similar to that of *MIR159c:GUS *(Figure 4b).

For *MYB120:GUS *and *mMYB120:GUS*, multiple transgenic lines were isolated, and GUS activity was observed exclusively in anthers (Figure 7e, f). No transgene expression was detected in vegetative tissues. *MYB120:GUS *and *mMYB120:GUS *showed indistinguishable anther specific expression patterns in inflorescences, both spatially and temporally, demonstrating that loss of the miR159 target site did not influence the expression pattern of *MYB120 *(Figure 7e, f). By comparison to *MYB101*, expression of *MYB120 *appeared to occur at earlier anther stages, but again included those stages in which *MIR159c:GUS *was expressed (Figure 4b).

### *MYB101 *and *MIR159c *have highly similar spatial and temporal transcriptional domains

Despite *MIR159c *(Figure 4b) and *MYB101 *(Figure 7) being both transcribed in an anther-specific manner in the inflorescence, miR159c is not the major cleavage regulator of *MYB101*, as demonstrated by the relative paucity of miR159 cleavage products recovered in *mir159ab*. However it has been found that in *Arabidopsis *roots, the *MIR395 *gene is transcribed in phloem companion cells, adjacent to xylem expressed targets, perhaps preventing 'leaky' target expression [40]. Similarly, to precisely determine if *MIR159c *and *MYB101 *are transcribed in adjacent cell types, and to investigate if *MYB101 *is subject to subtle miR159 regulation not detectable in whole anthers, transverse sections of GUS-stained *proMYB101:GUS*, *MYB101:GUS *and *mMYB101:GUS *anthers were examined using dark field microscopy.

**Figure 8:**
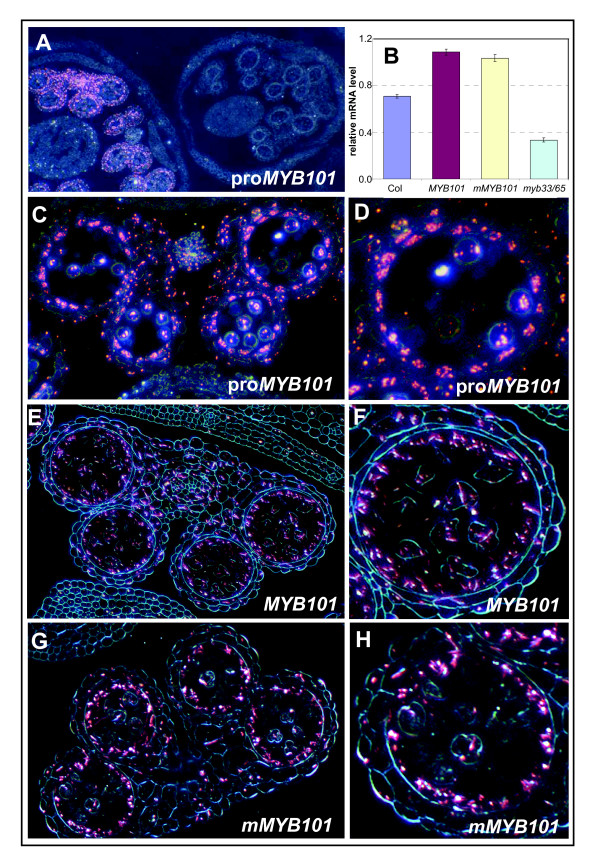
***MYB101 *and *mMYB101 *transgenes have indistinguishable expression patterns**. Anthers were stained overnight and embedded in paraffin. Transverse sections were examined by dark field microscopy. β-Glucuronidase (GUS) staining is shown by pink crystals. **(A) **Low magnification of *proMYB101:GUS *inflorescence showing *MYB101 *transcription is restricted to postmeiotic anthers. **(B) **Relative expression of *MYB101 *in wild type, *MYB101/mMYB101 *GUS lines and *myb33*.*myb65*. Analysis was performed on RNA extracted from inflorescences with measurements being the average of three replicates with error bars representing the standard error of the mean. mRNA levels are relative to cyclophilin. **(C) **GUS staining in *proMYB101*:*GUS *anthers. **(D) **Detail of a single locule of *proMYB101*:*GUS*. **(E) **GUS staining in *MYB101:GUS *anthers. **(F) **Detail of a single locule of *MYB101:GUS*. **(G) **GUS staining in *mMYB101*:*GUS *anthers. **(H) **Detail of a single locule of *mMYB101*:*GUS*.

Staining for all three *MYB101 *reporter transgenes appeared highly similar, with abundant GUS crystals appearing in the tapetum and to a lesser extent in the developing microspores, connective and other anther cell layers (Figure 8d-g). There was no obvious difference between staining in *MYB101:GUS *anthers, compared to *mMYB101*:*GUS *suggesting that miRNA regulation does not spatially or temporally delineate expression of MYB101 in anthers. The fact that the expression patterns of the *MYB101:GUS *and *mMYB101:GUS *transgenes do not differ from the *proMYB101*:*GUS *transgene, suggests that transcriptional controls of this gene predominantly determine its pattern of expression. Consistent with this, both *MYB101:GUS *and *mMYB101:GUS *transcript levels were at similar levels (Figure 8b), suggesting that the miR159 target site in *MYB101 *does not influence mRNA levels for this gene. However we cannot rule out the possibility that miR159 regulation results in subtle changes to MYB101 protein levels.

In a highly similar pattern to the *MIR159c:GUS *transgene, MYB101/mMYB101:GUS expression only occurs in postmeiotic anthers (Figure 8a), and although initial MYB101:GUS expression is found in many cell types, as development proceeds, expression becomes concentrated in the tapetum (Figure 8d). Also similar to *MIR159c:GUS*, degeneration of the tapteum coincides with the loss of GUS staining, explaining the transient MYB101:GUS expression in anthers. This demonstrated that *MIR159c *and *MYB101 *have overlapping transcriptional domains, both spatially and temporally, being both predominantly transcribed in postmeiotic tapetal cells, indicating that MYB101 expression should be subjected to miR159c regulation. Yet the fact that both MYB101 (Figure 8) and MYB33 [32] protein can accumulate in postmeiotic tapetal cells, implies that at most, miR159c could only be acting as a tuning miRNA.

The highly similar family members *MYB33 *and *MYB101 *are both expressed in anthers and both strongly in the tapetum. However *MYB33 *is expressed in young anthers, at premeiotic developmental stages, and in *myb33.myb65 *plants the block in anther development is premeiotic [32], before *MYB101 *is expressed. Consistent with this the *MYB101 *mRNA level is downregulated in *myb33.myb65 *flowers (Figure 8b), suggesting a developmental hierarchy of *MYB *expression in *Arabidopsis *anthers and a possible contributing factor to the lower *MYB101 *transcript levels observed in *35S*:*MIR159a *plants [29], and higher *MYB101 *transcript levels in *mir159ab *inflorescences (Figure 3d).

### Overexpression of *mMYB101 *does not result in anther/pollen defects

**Figure 9:**
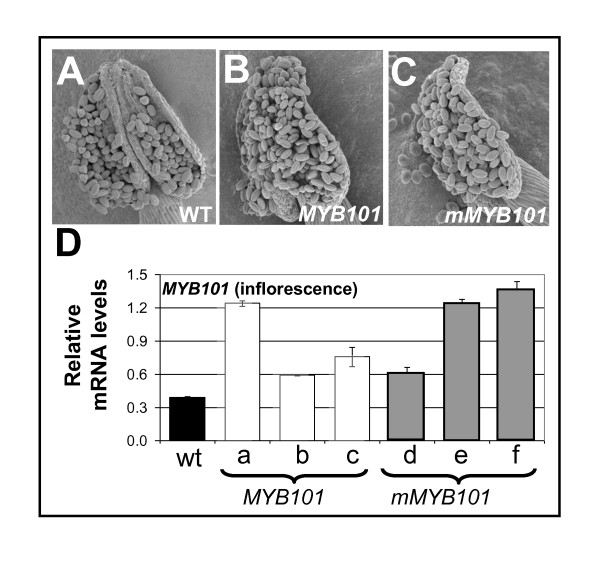
**Anthers and pollen of *MYB101 *and *mMYB101 *transgenic plants are indistinguishable from wild type**. Scanning electron micrographs of anthers/pollen from: **(A) **a wild-type plant, **(B) **a *MYB101 *transgenic plant, **(C) **a *mMYB101 *transgenic plant. **(D) **Real-time quantitative (qRT)-PCR analysis of *MYB101 *mRNA levels in inflorescences of wild type (WT) and *MYB101 *(lines A, B, C) and *mMYB101 *(lines D, E, F) transgenic lines. mRNA levels were normalised to cyclophilin, with measurements being the average of three replicates and error bars representing the standard error of the mean.

As the above expression analysis cannot rule out subtle miR159 regulation of *MYB101 *that has biological consequences, *MYB101*/*mMYB101 *transgenes were generated, where the GUS gene used in the previous analyses was replaced with 1162 bp of genomic sequences downstream of the *MYB101 *stop codon. Anthers and pollen were examined in *MYB101*/*mMYB101 *plants and in all cases were found to be indistinguishable from wild type (Figure 9a-c). Determination of total *MYB101 *transcript levels (endogenous plus transgenic *MYB101*) in inflorescences of *MYB101 *and *mMYB101 *transgenic plants found expression had increased 1.5-3-fold relative to wild type, confirming that the transgenic lines were overexpressing *MYB101 *(Figure 9d). This was also reinforced at the protein level, where the anthers and pollen of both *MYB101:GUS *and *mMYB101:GUS *lines that showed MYB101:GUS/mMYB101:GUS expression in anthers (Figure 8) were in all cases morphologically indistinguishable from wild type (Additional file 4, Figure S3), as the *MYB101:GUS *fusion protein had biological activity (see below).

**Figure 10:**
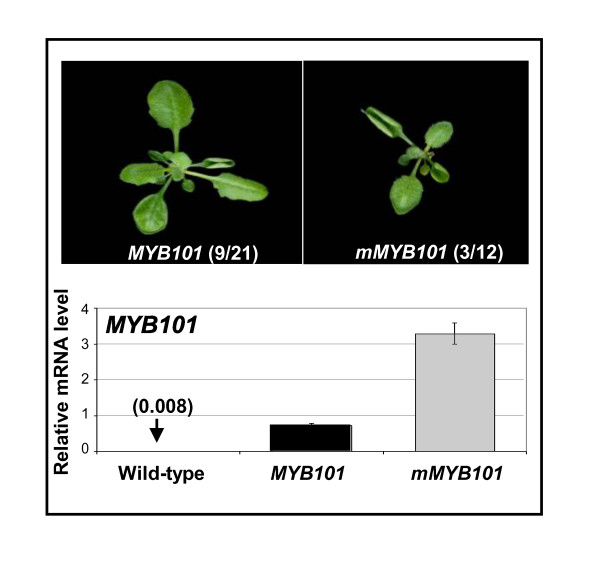
**Ectopic *MYB101*/*mMYB101 *expression can result in rosette phenotypes**. Aerial views of transgenic *MYB101 *and *mMYB101 *rosettes displaying leaf curling, with numbers in brackets indicating the frequency of the leaf curl phenotype. *MYB101 *transcripts from both *MYB101 *and *mMYB101 *leaf curl rosettes were assayed by real-time quantitative (qRT)-PCR. The numbers in brackets show the relative levels of *MYB101 *mRNA in wild-type rosettes. Analysis was performed on RNA extracted from rosettes with measurements being the average of three replicates with error bars representing the standard error of the mean. mRNA levels are relative to cyclophilin.

Although extensive genomic flanking sequences were used, a number of transgenic lines resulted in ectopic expression of *MYB101 *where mRNA levels in the rosette tissues were more than a 100-fold higher than in wild type, resulting in phenotypic characteristics similar to *mMYB33 *or *mir159ab *[14] plants (Figure 10). This ectopic expression implies these phenotypes must be transgenic artefacts, which is supported by genetic analysis, as *mir159ab*.*myb33*.*myb65 *rosettes have a wild-type appearance. This illustrates that expressing these *MYB *genes with such large flanking regions is unable to ensure transcription always faithfully mimics the endogenous gene.

Furthermore, ectopic *mMYB101/mMYB101:GUS *expression could also lead to rosette phenotypes similar again to *mir159ab *(data not shown), indicating that this transgene has biological activity, yet is unable to produce anther defects even under strong ectopic expression. Thus rendering *MYB101 *resistant to miRNA regulation and/or overexpression of this gene resulted in no obvious phenotypic consequences for anther or pollen development.

## Discussion

For many potential miR159 target genes, functional analysis has failed to identify any major morphological or molecular impact associated with miR159 regulation, despite more commonly taken approaches insinuating otherwise. The overlapping transcriptional domains of miR159c and many potential targets in anthers has identified a miR159c:target gene module, which we speculate may have once been important, but now appears to be quiescent in nature. We reason that the relatively recent demise of such a module may account for the conservation of miRNA target sites within potential target genes that appear principally independent of miR159 regulation. If correct, this would illustrate the evolutionarily fluid nature of miRNA:target relationships that exist even within ancient miRNA:target modules; while miR159a and miR159b are ingrained and critical for development, miR159c appears to be veering towards obsolescence. With respect to the targets, miR159 regulation of *MYB33 *is critical throughout the majority of the plant, while *MYB101 *expression appears principally independent of miR159 regulation. Therefore closely related members of the same gene family with conserved miRNA binding sites are regulated by very different mechanisms; *MYB33 *and *MYB65 *expression is largely determined post-transcriptionally, while *MYB101 *expression is predominantly determined at the transcriptional level. This case highlights the need for functional analysis of predicted miRNA targets, and that evidence from other methodologies may overestimate the extent of miRNA regulation that confers functional importance. These results reflect similar findings in metazoan systems; where among many potential targets, often only a single or few mRNAs may be relevant *in vivo *targets for a particular miRNA (reviewed in [41]). Thus a greater functional specificity of miRNAs than predicted by bioinformatics or molecular methodologies is an emergent and unifying theme in both plant and animal studies.

### *MIR159c *has the hallmarks of obsolesce

Firstly, *MIR159c *appears to be very weakly expressed; a fact that also appears to be the case in *A lyrata *and *Capsella rubella *[34]. The presence of a stretch of three transposable elements 214 bp upstream of the *MIR159c *stem-loop that are absent in the *A lyrata MIR159c *locus, indicates major sequence changes are tolerated at the *MIR159c *locus, possibly reflecting reduced selection pressure. In addition to low transcription of *MIR159c*, additional processing inefficiencies further reduce miR159c accumulation ([25]; Figure 3). Furthermore *mir159c *displayed no obvious phenotypic defects or further redundancy with *mir159ab *(Figure 1). Finally, no potential targets of miR159c showed any deregulation in *mir159c *(Figure 5). Together, these analyses have failed to find any obvious functional role for *MIR159c*.

The only indication *MIR159c *may have any activity was the complementation of *mir159ab *by a *35S:MIR159c *transgene (Figure 3). This demonstrates that when transcribed at sufficiently high levels, it is still able to effectively silence *MYB33 *and *MYB65*; hence *MIR159c *could not yet be considered a pseudogene. This raises the possibility that the gene, if induced, may play a role in controlling *MYB *activity. However this would seem unlikely in rosette or inflorescence tissues. In the rosettes miR159a and miR159b are so high a *35S:MIR159c *transgene has no effect. In the inflorescence, although the *35S:MIR159c *transgene could reduce *MYB *transcript levels in the *mir159ab *background, it could not repress *MYB *expression to levels that could cause male sterility. Therefore it is likely that not only would *MIR159c *transcription need to increase, but also its poor processing efficiency [25] be overcome for it to be highly expressed to exert a biological outcome.

Evidence suggests that *MIR159c *may have previously carried out a specialised function that has now become obsolete. In contrast to *MIR159a *and *MIR159b *that are broadly expressed, *MIR159c *appears to be transcribed specifically in the inflorescence in the same discrete anther cell layer (the tapetum) as *MYB101 *(Figure 8) and *MYB33 *[32]. It is tempting to speculate that *MIR159c *had previously subfunctionalised and corresponded to a discrete regulatory module, controlling *GAMYB *expression in anthers compared to the broader *MIR159a *and *MIR159b *module controlling *MYB33 *and *MYB65 *in vegetative tissues. Precedence for this is that *GAMYB *expression in rice anthers appears under miR159 control [42], a role miR159c could have performed in *Arabidopsis *if its activity had been stronger; as most of the *Arabidopsis GAMYB-like *genes are strongly transcribed in anthers [39]. However in *Arabidopsis *this requirement no longer appears important for two reasons. Firstly, from the perspective of *MIR159c*, loss of this gene has no noticeable molecular or phenotypic consequence (Figures 1, 2, 5 and 6). Secondly, from the perspective of potential miR159c targets, not only does *MYB101 *and *MYB120 *expression appear independent of miR159 regulation, overexpression of a *mMYB101 *transgene results in no obvious detrimental anther phenotype (Figure 9). By contrast, *GAMYB *overexpression in transgenic barley results in male sterility [43], demonstrating that modulation of *GAMYB *levels is required. Therefore the level of anther *GAMYB *and its regulation by miR159 appears important in cereals, and may reflect that miR159c regulation of *MYB101 *may represent a former important regulatory module for anther development that is now obsolete in *Arabidopsis*.

However these conclusions that are in part based on a lack of demonstrated function cannot be definitive, as we are unable to rule out that in a certain cell type or environmental condition, miR159c or the miR159 binding site within *MYB101 *and the other targets may have a functional role. Nevertheless, even if this is the case for some of these targets, our functional analysis of the entire miR159 family generally supports the notion of a much narrower functional specificity of plant miRNAs, rather than the possibility that they exert their effects through a broad range of targets.

### Diverse modes of regulation of closely related genes resulting in similar expression outcomes

Despite *MYB101 *having a highly conserved miR159 binding site, detailed analysis in inflorescences of *MYB101:GUS *and *mMYB101:GUS *transgenic plants failed to find any miR159 regulation that delineates the expression of this gene (Figure 8). Furthermore, *MYB101 *and *mMYB101 *transgenes, when expressed in their native domain in anthers, had no apparent impact on development (Figure 9). Therefore in stark contrast to *MYB33 *[34], miR159 appears peripheral to regulation of *MYB101*.

Curiously *MYB33 *and *MYB101 *represent two closely related genes that are predominantly expressed in seeds and anthers [32,44], but via two very different mechanisms. *MYB33 *is almost constitutively transcribed throughout the plant, only to be silenced by miR159 except in seeds and anthers [32], hence post-transcriptional regulation delineates the final expression pattern of the MYB33 protein. Conversely, *MYB101 *is specifically transcribed in seeds and anthers, tissues in which miR159 activity appears weak or absent [32] implying transcriptional regulation of this gene predominantly determines where the MYB101 protein is expressed. Thereby these two very closely related gene family members have very different regulatory relationships with miR159, as defined by their transcriptional domains.

### Limitations of experimental validation of miRNA targets

This study has illustrated the limitations of several current methodologies used in miRNA target gene identification. Firstly, 5'-RACE recovery of miRNA-guided cleaved mRNA targets has been considered the gold standard for determining whether a particular mRNA is an authentic miRNA target [4]. However, although miR159-guided cleavage products for *MYB101 *in inflorescences were recovered, it appears that there is no obvious functional role of this miRNA-target relationship. It should be noted that while *MYB33 *cleavage products were observable after the first round of PCR, this was not the case for any other *MYB *target genes. This may suggest that the abundance of cleavage is orders of magnitude less for these other genes when compared to *MYB33*. This agrees with degradome data that has found *MYB33 *and *MYB65 *were miR159 regulated, but not *MYB81*, *MYB101*, *MYB120 *or *DUO1 *[8-10]. This could reflect the extremely sensitive nature of the 5'-RACE assays, where nested PCR and gel purification of the expected size band would detect basal background miRNA activity of no functional consequence. Therefore our findings here and previously [14] concur with the quantitative degradome approaches that will predominantly detect strong regulatory relationships [9].

Secondly, analysis of phenotypical changes resulting from disruption of miRNA binding sites in putative mRNA targets has been used to identify the functional importance of miRNA-mediated regulation. However the results here show that transgenic expression can potentially misrepresent the extent of miRNA regulation for a particular target, even when transcribed under its native promoter (Figure 10). This supports the notion that inferring miRNA regulation based on transgene expression can be misleading [45].

Finally overexpression of miRNAs may misrepresent what genes are miRNA regulated *in vivo*. By example, it has been reported that mRNA levels of *MYB33 *and *MYB65 *were not downregulated by a *35S:miR159a *transgene, but rather *MYB101 *was the most strongly downregulated gene [29]. However the fact that *35S:miR159a *anthers were sterile, similar to the *myb33.myb65 *mutant, suggests that the downregulation of *MYB101 *could be in part a secondary effect of *MYB33 *and *MYB65 *silencing. This was substantiated by the observation that *MYB101 *levels are considerably lower in *myb33.myb65 *flowers (Figure 8b).

## Conclusions

As many evolutionary changes are thought to occur through alterations to gene regulation, this will also include miRNA:target regulatory modules becoming obsolete. The hypothesis of frequent birth and death of evolutionary non-conserved miRNAs [46] can now be extended to include conserved miRNAs such as miR159. The remnants of these systems, which include both the *MIRNA *genes and the binding motifs in target mRNAs, may be leading to the overestimation of the complexity of miRNA regulation that confers a functional impact.

## Methods

### Isolation and genotyping of *mir159c*

The SAIL_248_G11 T-DNA mutant was found on the SIGnAL 'T-DNA Express' *Arabidopsis *Gene Mapping Tool [36]http://signal.salk.edu/cgi-bin/tdnaexpress and ordered from the *Arabidopsis *Biological Resource Center. Amplification using gene specific primers (Additional file 5, Table S1) detected the wild-type allele, and amplification using the T-DNA specific primer LB3 and *MIR159c *gene specific primers (Additional file 5, Table S1) isolated the T-DNA border junctions.

### RNA analysis

Total RNA was extracted from inflorescences using TRIzol reagent (Invitrogen; http://www.invitrogen.com/) with the following modifications to the manufacturers protocol. (1) The chloroform extraction was repeated. (2) Precipitation of RNA was carried out overnight at -20°C. (3) Samples were heated only to 37°C after dissolving in nuclease free water. RQ1 DNAse (Promega; http://promega.com/) was used to treat RNA except for qSL-PCR (see below), where no DNase treatment was carried out. RNA was cleaned using Plant RNAeasy columns (Qiagen; http://www.qiagen.com/). cDNA synthesis was carried out using Superscript III reverse transcriptase (Invitrogen) according to the manufacturers protocol with an oligo dT primer (Invitrogen). For each RNA sample, three separate cDNA synthesis reactions were carried out. Real-time quantitative (qRT)-PCR was carried out as described in Allen *et al*. [14]. Primers used for this analysis are described in Additional file 5, Table S2.

### Histochemical analysis of GUS activity

*In situ *GUS activity staining was performed using the method of Jefferson *et al*. [47]. Tissues were transferred to this reagent in 1.5 ml Eppendorf tubes, vacuum infiltrated for 2 min, and left overnight or as described elsewhere at 37°C. Afterwards, stained tissues were rinsed three times using 70% ethanol. For preparation of GUS-stained anther sections for light microscopy, inflorescences were stained in GUS reagent for 48 h at 37°C and dehydrated in a graded ethanol series (70%, 95%, 100%). Inflorescences were then infiltrated and embedded with LR white resin (London Resin Company, London, U.K). Transverse sections (2 μm) were made with a Leica Ultracut 6 ultramicrotome (Leica UK; http://www.leica.com/). Sections were stained with 1% toludine blue for 1 min.

### Images

Digital photographs of rosettes, siliques and whole plants were taken at the CSIRO Phytotron studio, Canberra, Australia. Scanning electron microscopy of stamens, anthers, pollen and seed was performed by gold-coating tissues using a high resolution splutter coater (Bio-Rad; http://www.bio-rad.com/), and examination with a Cambridge S360 scanning electron microscope (SEM) (Cambridge, UK). Images of GUS-stained anther sections were taken with a Leica DMR upright microscope for dark-field microscopy. Images of GUS-stained seedlings, inflorescences, and individual flowers were taken with a Leica MZFLIII dissecting microscope.

### Quantitative stem-loop qRT-PCR miRNA analysis

For TaqMan stem-loop qRT-PCR miRNA analysis (qSL-PCR), RNA was prepared using TRIzol as described above for expression analysis. For the assays, Applied Biosystems ABI TaqMan MicroRNA quantitative real-time stem-loop PCR assays were used http://www.appliedbiosystems.com/, and the manufacturers instructions were followed with the following modifications. (1) For each RNA sample, there were three stem-loop cDNAs made, and the reverse transcriptase (RT) step was multiplexed using both *sno101 *RT primer and miR159a, miR159b or miR159c primer. (2) The cDNA (15 μl) was diluted with 86.4 μl of nuclease free water, so that 9 μl of RT reaction could be pipetted into 20 μl total PCR reaction volume. (3) Each cDNA was assayed in triplicate on a Corbett real-time PCR machine (Corbett, http://www.corbettlifescience.com/). Primer sequences for qSL-PCR are proprietary to Applied Biosystems. Expression of miR159 was normalised to *sno101*, using the comparative concentration analysis program of rotor gene software (Corbett).

### Generation of binary vectors and transgenic plants

To generate the 2 × *35S:MIR159c *construct 1226 bp of *MIR159c *sequence comprising 525 bp upstream and 485 bp downstream of the *MIR159c *stem loop was amplified from *Arabidopsis *and cloned into the vector pMDC32 (Invitrogen). To generate the *MIR159c:GUS *construct 1.3 kb of genomic sequence immediately upstream of the *MIR159c *stem loop was cloned into the vector pBI 101.1. For generation of the *MYB101 *genomic construct 6333 bp of *MYB101 *genomic sequence comprising upstream and downstream genomic regions were amplified from *Arabidopsis *genomic DNA. miRNA resistant target sites were introduced by PCR to generate the construct *mMYB101*. The genomic fragments were cloned into pMDC99 [49 For *MYB101 *and *MYB120 *GUS constructs, genomic sequences of 5171 and 3128 bp, respectively were amplified, for MYB101 GUS, this sequence was identical to the genomic fragment used for the genomic construct above except there was no genomic sequence beyond the stop codon. miRNA resistant versions were produced by PCR, and both *MYB101/120 *and *mMYB101/120 *fragments were cloned into the vector pMDC164 [48]. All vectors were transformed into *Agrobacterium *and *Arabidopsis *as previously described [14].

### Modified 5'-RACE of cleaved miRNA targets

mRNA was purified from the same inflorescence RNA samples used for qRT-PCR analysis of miR159 targets in wild type and *mir159ab *[14], using 100 μg of total RNA. A Gene-Racer kit (Invitrogen) was used for 5'-RACE, except the decapping protocol was not carried out, and the adapter was ligated directly to mRNA. PCR of *MYB33 *and *MYB101 *sequence downstream of the miR159 cleavage site was used as a control to check 5' cDNA amplification was successful. The products from the second (nested) round of 5'-RACE were gel purified using a Wizard preps PCR purification kit (Promega) and ligated into pGEM-T easy (Promega). Plasmids were transformed into *Escherichia coli *XL-10 gold and purified. Clones were digested with *Not*I to verify they contained inserts of the correct size, and were sequenced. Primers used for this analysis are shown in Additional file 4, Table S3.

## Competing interests

The authors declare they have no competing interests.

## Authors' contributions

RSA conceived and carried out the majority of the experiments and drafted the manuscript. JL contributed Figures 4a, b, d, 5f, g, 6a, b, e, f (with assistance from MMA-P) and 7d-f and helped draft the manuscript. RGW assisted with sectioning of anthers and supervision of the project. FG supervised the project. AAM supervised the project and helped draft the manuscript. All authors read and approved the final manuscript.

## Supplementary Material

Additional File 1 **miR159 targets predicted by bioinformatics or miR159 overexpression, or validated by rapid amplification of 5' complementary DNA ends (5'-RACE) or degradome analysis**. . All predicted miR159 targets from three different plant bioinformatics programs and verified miR159 targets from published 5'-RACE, degradome and overexpression studies; overexpression describes targets shown to have lower RNA levels than wild type in 35S:MIR159a transgenic plants. Mature miR159 members are shown 3'-5'. Target mismatches with miR159a are bold. Bioinformatically identified targets specific for miR159b or miR159c are indicated with brackets. Anther/pollen expression data is compiled from genevestigator [39] . Not all genes were available on the dataset (shown as NA).  D = degradome; H = RNAhybM [28]; M = miRU [49]; OE = overexpression; P = plant small RNA target [50]; R = 5'-RACE.  Y = yes, N = No. Click here for file

Additional File 2 **Figure S1**. Scanning electron microscopy of pollen from **(a) **wild type, **(b) ***35S:MIR159c *in *mir159ab *(line 2), **(c) ***35S:MIR159*c in wild type, and **(d) **germinating pollen of *35S:MIR159c *in *mir159ab *(line 2). Scale bars are 20 μM. Click here for file

Additional File 3 **Figure S2**. Control amplification of adapter ligated rapid amplification of complementary DNA ends (RACE) cDNA. The gene racer RNA oligonucleotide was ligated to wild-type (col) and *mir159ab *total inflorescence RNA, and control real-time (RT)-PCR amplifications were carried out using primers downstream of the miR159 site. Genomic DNA was also amplified using the same conditions using the *MYB81 *specific primers. Click here for file

Additional File 4 **Figure S3**. Microscopy of flowers and scanning electron microscopy of anthers/pollen of *MYB101/mMYB101:GUS *lines. Flowers, anthers and pollen from all *MYB101/mMYB101:GUS *lines were examined and found to be morphologically indistinguishable from wild type (Figure 9). Click here for file

Additional File 5 **Tables S1-S3**. Primers used in this study. Click here for file
