# Supplementary material for: MicroR159 regulation of most conserved targets in Arabidopsis has negligible phenotypic effects
Source: Silence. 2010 Oct 28;1:18. doi: 10.1186/1758-907X-1-18 (PMC2988730; doi:10.1186/1758-907X-1-18)
Supplement: Additional File 1 — miR159 targets predicted by bioinformatics or miR159 overexpression, or validated by rapid amplification of 5' complementary DNA ends (5'-RACE) or degradome analysis. . All predicted miR159 targets from three different plant bioinformatics programs and verified miR159 targets from published 5'-RACE, degradome and overexpression studies; overexpression describes targets shown to have lower RNA levels than wild type in 35S:MIR159a transgenic plants. Mature miR159 members are shown 3'-5'. Target mismatches with miR159a are bold. Bioinformatically identified targets specific for miR159b or miR159c are indicated with brackets. Anther/pollen expression data is compiled from genevestigator [39] . Not all genes were available on the dataset (shown as NA). D = degradome; H = RNAhybM [28]; M = miRU [49]; OE = overexpression; P = plant small RNA target [50]; R = 5'-RACE. Y = yes, N = No. [file 1758-907X-1-18-S1.PDF]

Target	At identity	Sequence vs miR159	Prediction/validation	Anther/pollen prevalent?
		(a) AUCUCGAGGGAAGUUAGGUUU		
		(b) UUCUCGAGGGAAGUUAGGUUU		
		(c) UCCUCGAGGGAAGUUAGGUUU		
MYB33	At5g06100	U**G**GAGCUCCCUUCA**U**UCCAA**U**	P/M/H/R[20]/D[8-10]	Y
MYB65	At3g11440	U**G**GAGCUCCCUUCA**U**UCCAA**U**	P/M/H/R[20]/D[8-10]	Y
MYB101	At2g32460	UAGAGCU**U**CCUUCAA**A**CCAAA	P/M/H/R[25, 27-28]/OE[29]	Y
MYB104	At2g26950	U**G**GAGCUCCCUUCA**U**UCCAA**G**	P/M/H	Y
MYB97	At4g26930	**AU**GAGCUC**U**CUUCAA**A**CCAAA	P/M/H	Y
MYB120	At5g55020	**AGC**AGCUCCCUUCAA**G**CCAAA	P/M/H/OE[29]	Y
MYB81	At2g26960	U**C**GAG**U**UCCCUUCA**U**UCCAA**U**	M/H	Y
DUO1	At3g60460	U**G**GAGCUCC**A**UUC**G**AUCCAAA	P/M/R[25, 28]	Y
TCP2	At4g18390	**AG**G**G**G**GA**CCCUUCA**G**UCCAA**U**	M(159c)	N
ACS8	At4g37770	U**C**GAG**U**U**U**C**U**UUCAAUCCAAA	P/M/R[28]/OE[29]	N
OPT1	At5g55930	UAGAGCU**UU**CUUCA**U**UCCAA**C**	M/H/R[29]/OE[29]	Y
Zinc/Cu SODM	At5g18100	U**G**GA**C**CUC**A**CUUCAAUCCA**UU**	D[10]	N
MRG1	At2g34010	UAGAGC**C**CCCUUCAA**A**CCAAA	P/H/R[28]/D[10]	NA
MRG-similar	At1g29010	UAGAGC**CU**CCUUCAA**G**CCAAA	P/M	NA
PHD(ATX3)	At3g61740	UAGAGCUC**U**CUU**A**A**G**UC**U**AAA	P/M	Y
Anion/ExProt	At3g06450	**A**AGAGCUCC**G**UUCA**G**UCCA**CG**	M	N
NPH3 Prot	At5g17580	**A**A**A**AGCU**U**CCU**A**C**G**AUCCAA**G**	M (159b)	N
NAS2	At5g56080	UAGAGCU**UU**CUU**GU**AUCCAA**U**	M	N
SPL	At4g27330	**U**<br>A**U**GAGCUC**U**CUUCAAUCCAAA	H	Y
PPDK	At4g15530	**G**<br>**A**AGAG**U**U**U**CCUUCAAUCCAAA	P	Y
