# Supplementary figures and images for: MicroR159 regulation of most conserved targets in Arabidopsis has negligible phenotypic effects
Source: Silence. 2010 Oct 28;1:18. doi: 10.1186/1758-907X-1-18 (PMC2988730; doi:10.1186/1758-907X-1-18)

Figure S1

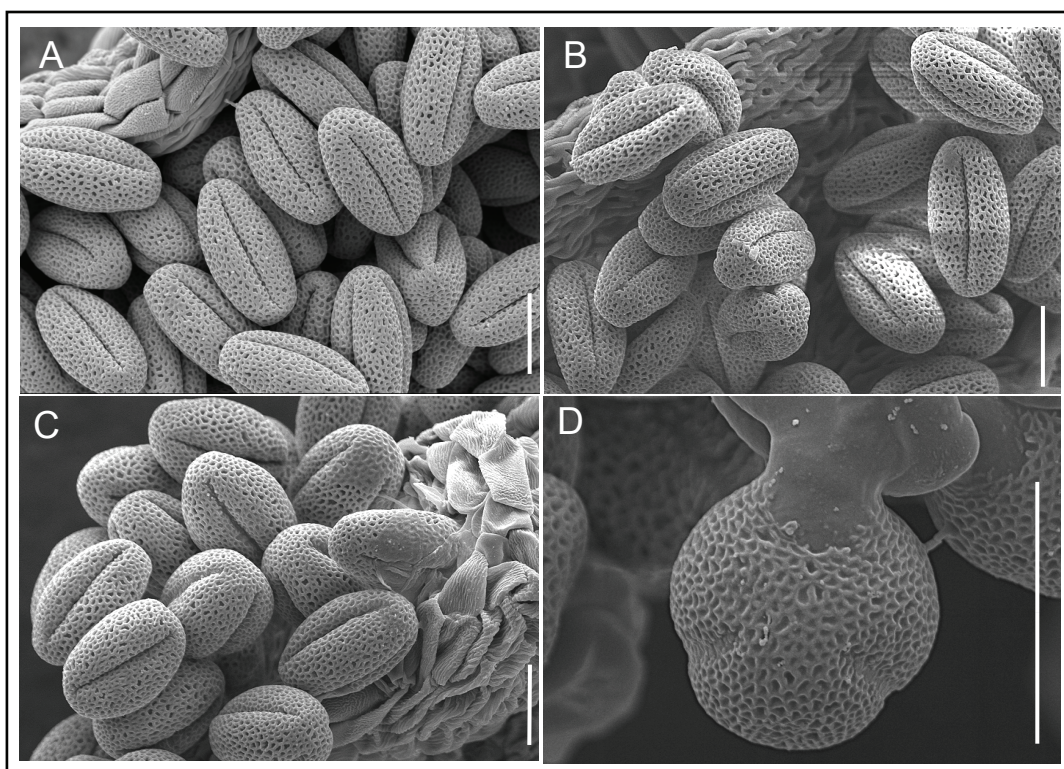

Supplement: Additional File 2 — Figure S1. Scanning electron microscopy of pollen from (a) wild type, (b) 35S:MIR159c in mir159ab (line 2), (c) 35S:MIR159c in wild type, and (d) germinating pollen of 35S:MIR159c in mir159ab (line 2). Scale bars are 20 μM. [file 1758-907X-1-18-S2.PDF]

Figure S2

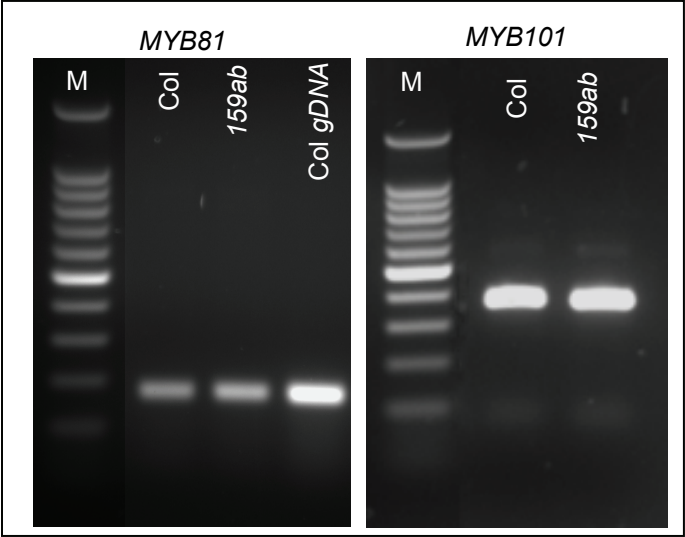

Supplement: Additional File 3 — Figure S2. Control amplification of adapter ligated rapid amplification of complementary DNA ends (RACE) cDNA. The gene racer RNA oligonucleotide was ligated to wild-type (col) and mir159ab total inflorescence RNA, and control real-time (RT)-PCR amplifications were carried out using primers downstream of the miR159 site. Genomic DNA was also amplified using the same conditions using the MYB81 specific primers. [file 1758-907X-1-18-S3.PDF]

Figure S3

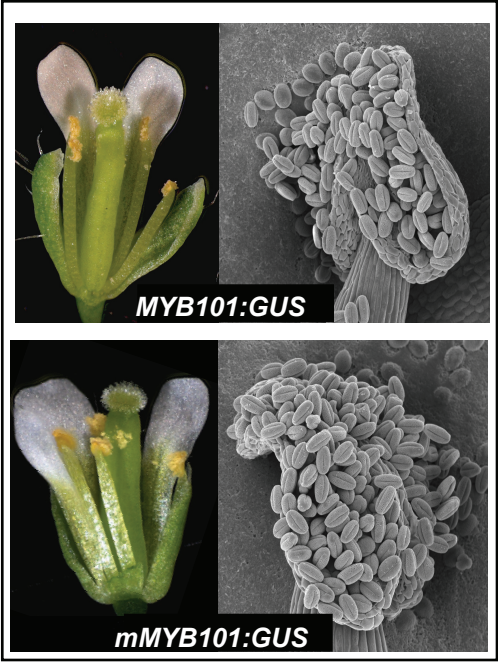

Supplement: Additional File 4 — Figure S3. Microscopy of flowers and scanning electron microscopy of anthers/pollen of MYB101/mMYB101:GUS lines. Flowers, anthers and pollen from all MYB101/mMYB101:GUS lines were examined and found to be morphologically indistinguishable from wild type (Figure 9). [file 1758-907X-1-18-S4.PDF]
