# Supplementary material for: MicroR159 regulation of most conserved targets in Arabidopsis has negligible phenotypic effects
Source: Silence. 2010 Oct 28;1:18. doi: 10.1186/1758-907X-1-18 (PMC2988730; doi:10.1186/1758-907X-1-18)
Supplement: Additional File 5 — Tables S1-S3. Primers used in this study. [file 1758-907X-1-18-S5.DOC]

**Supplementary tables S1-S3**

**(S1)Primers for Genotyping of *mir159c***

| **primer name** | **Sequence (5’->3’)** |
| --- | --- |
| *MIR159c* 5’ Forward | ATACTCCATAAACCATAGTGTTGCACCCT |
| *MIR159c* 3’ Reverse | TTCAAATTGGCACACGAACGTCTTCTCGT |
| LB3 (T-DNA) | TAGCATCTGAATTTCATAACCAATCTCGATACA |

**(S2) Primers for gene expression analysis**

| **gene** | **Primer Forward (5’->3’)** | **Primer Reverse (5’->3’)** |
| --- | --- | --- |
| *MYB33*(At5g06100) | TCGTCATCTCCTCCACACTCTG | CCTCGGATTTAGTTTGGGATAC |
| *MYB65*(At3g11440) | CTTCCCCAAAGCAAATCTG | TTCACTGCCCCAAACAAG |
| *MYB101*(At2g32460) | CGAGTTCTTTCCCTTTAGGACT | TGGCTCATTGTACTTGTTGTG |
| *MYB81* (At2g26960) | AACACTTTGGTTCAATCTCCTCTG | ATGACTGAAACAGTGAAGATTCTG |
| *MYB97* (At4g26930) | GGTTTGCATACAAATACCTGTCAG | GTTGTTGTTGTTGTTGTTGTCCTC |
| *MYB104*(At2g26950) | GCAGAACAATATAACCCAATGCTG | ATGATGATGGGAAATCTGTTGGTG |
| *MYB120* (At5g55020) | AACTTCACAGACAACGAGAGACAG | TCGGAAGAGAAGCTGTGAGTTGTC |
| *MYB125/DUO1*(At3g6046) | TGTGAAGAATTTCTGGAGTAGCAG | AGAGGATTGACGGATTGGTTTGAC |
| *Cyclophilin*(At2g2996) | TGGACCAGGTGTACTTTCAATGG | CCACTGTCTGCAATTACGACTTTG |
| *MIR159c* (At2G46255.1) | AGCTCCTTTTCTTCTTCTCTTAAT | CGTCTTCTCGTAAATAAACAACATT |

**(S3) Primers For 5’- RACE of miR159 targets**

| **gene** | **1^st^ round primer (5’->3’)** | **2^nd^ round primer (5’->3’)** |
| --- | --- | --- |
| *MYB33* | CTCCTCGGATTTAGTTTGGGATACAGTAACCTG | GGCTTCCAGAAGCAACATATCGAGCAGACCAG |
| *MYB101* | GTTTTGCATTTCTGTCACCCGATACCAATCAG | CTAACGTCCTTGAAGAGTCCGCCTCGAGAC |
| *MYB81* | CAGTGAAGATTCTGAATCGGTATCGGTTGC | CAATCTGATGGTGTTTGAGCTGTCAGAGGAG |
| *MYB97* | GTTGTTATCACAGAGGATTGCCCGTTTGAG | GTTGTCCTCAAGCGCAGCCATGATTTGTCTC |
| *MYB104* | GAATGATGATGGGAAATCTGTTGGTGTCTG | GGTATCGGTTTTCGGTATTTCTCCTAATGAACC |
| *MYB120* | GTTGTGGTTCGGAAGAGAAGCTGTGAGTTGTC | CGGAACTGTTAAGGTTATGATTCTGTCTCTCG |
| *DUO1* | CCCTGAGAGGATTGACGGATTGGTTTGACG | GACAACTCACAGCTTCTCTTCCGAACCACAAC |
